# *In Vivo* Transplantation of Enteric Neural Crest Cells into Mouse Gut; Engraftment, Functional Integration and Long-Term Safety

**DOI:** 10.1371/journal.pone.0147989

**Published:** 2016-01-29

**Authors:** Julie E. Cooper, Conor J. McCann, Dipa Natarajan, Shanas Choudhury, Werend Boesmans, Jean-Marie Delalande, Pieter Vanden Berghe, Alan J. Burns, Nikhil Thapar

**Affiliations:** 1 Stem Cells and Regenerative Medicine, UCL Institute of Child Health, 30 Guilford Street, London, United Kingdom; 2 Laboratory for Enteric NeuroScience, Translational Research Center for GastroIntestinal Disorders, Department of Clinical and Experimental Medicine, University of Leuven, Leuven, Belgium; 3 Centre for Digestive Diseases, Blizard Building, Barts & The London School of Medicine & Dentistry, Queen Mary, University of London, London, United Kingdom; 4 Department of Clinical Genetics, Erasmus MC University Medical Centre, Rotterdam, The Netherlands; 5 Department of Gastroenterology, Great Ormond Street Hospital NHS Foundation Trust, Great Ormond Street, London, United Kingdom; Temple University School of Medicine, UNITED STATES

## Abstract

**Objectives:**

Enteric neuropathies are severe gastrointestinal disorders with unsatisfactory outcomes. We aimed to investigate the potential of enteric neural stem cell therapy approaches for such disorders by transplanting mouse enteric neural crest cells (ENCCs) into ganglionic and aganglionic mouse gut *in vivo* and analysing functional integration and long-term safety.

**Design:**

Neurospheres generated from yellow fluorescent protein (YFP) expressing ENCCs selected from postnatal *Wnt1-cre;R26R-YFP/YFP* murine gut were transplanted into ganglionic hindgut of wild-type littermates or aganglionic hindgut of *Ednrb*^*tm1Ywa*^ mice (lacking functional endothelin receptor type-B). Intestines were then assessed for ENCC integration and differentiation using immunohistochemistry, cell function using calcium imaging, and long-term safety using PCR to detect off-target YFP expression.

**Results:**

YFP+ ENCCs engrafted, proliferated and differentiated into enteric neurons and glia within recipient ganglionic gut. Transplanted cells and their projections spread along the endogenous myenteric plexus to form branching networks. Electrical point stimulation of endogenous nerve fibres resulted in calcium transients (F/F0 = 1.16±0.01;43 cells, n = 6) in YFP+ transplanted ENCCs (abolished with TTX). Long-term follow-up (24 months) showed transplanted ENCCs did not give rise to tumours or spread to other organs (PCR negative in extraintestinal sites). In aganglionic gut ENCCs similarly spread and differentiated to form neuronal and glial networks with projections closely associated with endogenous neural networks of the transition zone.

**Conclusions:**

Transplanted ENCCs successfully engrafted into recipient ganglionic and aganglionic gut showing appropriate spread, localisation and, importantly, functional integration without any long-term safety issues. This study provides key support for the development and use of enteric neural stem cell therapies.

## Introduction

Enteric neuropathies represent some of the most severe clinical gastrointestinal (GI) disorders and are characterised by failure of normal propulsive contractile activity leading to functional obstruction to the flow of luminal contents.[1–3] Archetypal disorders include the congenital disorder Hirschsprung disease and acquired oesophageal achalasia, which result from an absolute absence or loss of intrinsic enteric neurons in the distal and proximal parts of the GI tract respectively. More subtle neuronal defects underlie other disorders such as intestinal pseudo-obstruction and slow transit constipation, which show evidence of neuropathy on histology or on contractile profiles from intestinal physiological studies.[4–7]

Enteric neuropathies are generally devastating conditions, which left untreated are life threatening. Current available treatments are largely limited to surgery to decompress the intestine or resect its most abnormal segments and the provision of specialised nutrition, often parenteral, to preserve growth and development. Outcomes of such interventions are often unsatisfactory and associated with significant complications[8, 9] highlighting the need for alternative approaches.

Tremendous advances in regenerative medicine have brought the prospect of neural regeneration to replace deficient or defective enteric neurons as a potential therapeutic strategy for enteric neuropathies to the forefront.[8, 10–12] Several cell types have been identified as potential sources of donor cells for a cell replacement therapy, such as skin-derived precursors and central nervous system progenitors [13–16]. Arguably the most promising progress towards this ultimate goal has been made using enteric neural crest cells (ENCCs) which comprise enteric neural stem cells, neurons and glia and are derived from the original population of migratory neural crest cells (NCC) that, during embryogenesis, colonise the gut to form the enteric nervous system (ENS) [17–22].

The isolation of enteric neural stem cells was first described over a decade ago from murine gut, including models of Hirschsprung disease.[23] Since then there has been steady progress emanating from the isolation of such cells from human intestine including from the gut mucosa of patients obtained by conventional endoscopy[24]. We and others have shown that ENCCs contained within discrete cellular aggregates or neurospheres (including enteric neural stem cells, neurons and glia) formed in culture can colonise recipient bowel *in vitro*.[23–32].

Most recently, *in vivo* studies have confirmed that ENCCs, predominantly from embryonic mouse gut, are capable of migration, proliferation and differentiation and they retain some functionality after transplantation into postnatal mouse gut.[16, 33–36] Although these initial studies have addressed aspects of feasibility of ENS stem cell transplants, a number of challenges remain, including questions concerning long-term safety and the ability of transplanted cells to form neural networks, which connect to the endogenous enteric neural network. The ability to form an integrated and functional ENS after transplantation, *in vivo*, will be critical in the treatment of both aganglionic and euganglionic enteric neuropathies.

In this paper we confirm that postnatal mouse-derived ENCCs can colonise ganglionic mouse gut *in vivo* and form neural networks, which functionally integrate with endogenous ENS. We show that ENCCs similarly colonise aganglionic bowel. The transplanted cells do not appear to pose any long-term safety risk within the recipient animals.

## Materials and Methods

### Animals

Animals used for this study were maintained and the experiments performed were in accordance with the local approvals and the UK Animals (Scientific Procedures) Act 1986 under licence from the Home Office (PPL70/7500). *Wnt1-cre;R26R-YFP/YFP* mice[37–39] in which NCC express yellow fluorescent protein (YFP) were used to obtain YFP+ enteric NCC (ENCCs). *R26R-YFP/YFP* littermates (in which YFP is not expressed in the absence of cre) were used as ganglionic recipient bowel into which YFP+ ENCCs were transplanted. *Ednrb*^*tm1Ywa*^ mice lacking endothelin receptor type B (a model for Hirschsprung Disease) (obtained from Jackson Laboratory (JAX#003295)) were used as aganglionic recipient bowel for YFP+ ENCCs.

### Isolation, cell sorting and culture of ENCCs

YFP+ ENCCs were obtained from early postnatal (P2-P4) *Wnt1-cre;R26R-YFP/YFP* mice. Muscle strips of bowel (containing muscle layers and myenteric and submucosal plexuses) were dissociated enzymatically (Collagenase, Sigma, 1mg/ml) for 30 minutes at 37°C. Dissociated cells were washed with neurosphere medium (NSM) (DMEM F12 supplemented with B27 (InvitrogenLife Technologies, UK), N2 (Life Technologies, UK), 20ng/ml EGF (Peprotech, UK), 20ng/ml FGF (Peprotech, UK), and Primocin antibiotic (Invivo Gen, UK)) and filtered through a 100μm and 40μm mesh.

ENCCs were isolated from the total cell population using fluorescence activated cell sorting (FACS) for YFP. Dissociated cells were resuspended in NSM with 2% foetal calf serum before undergoing FACS using a MoFloXDP cell sorter (Beckman Coulter).

ENCC were washed with NSM before plating on fibronectin-coated wells of 6-well dishes in NSM. They were maintained in culture and generated neurospheres from around 1 week. Primary neurospheres generated in cultures maintained for a maximum of 30 days were used for the transplants.

### Transplantation of ENCCs into gut in vivo

YFP expressing ENCCs derived from *Wnt1-cre;R26R-YFP/YFP* mice were transplanted into the ganglionic distal colon of ‘wild-type’ *R26R-YFP/YFP* littermates (which did not express YFP in the absence of cre) via laparotomy at weaning (postnatal day 21) (n = 66). The same YFP expressing ENCCs were transplanted into Ednrb^tm1Ywa^ mutants (identified by their piebald markings) as early as practically possible at postnatal day 9–13 (n = 5).

Laparotomy was conducted under isoflurane anaesthesia, using 0.05% carprieve (Norbrook) and 0.25% marcaine polyamp (AstraZenica) analgesic agents. An incision was made in the lower abdomen through the peritoneum to reveal the bowel. The most distal portion of colon accessible from within the peritoneal cavity (around 0.5cm from the rectum), was exposed. ENCCs, as neurospheres (3 per animal), were selected using a fine glass capillary attached to a mouth pipette and inserted into a small pocket in the serosa of the bowel using the bevel of a syringe needle. The exteriorised bowel was returned to the abdomen and the laparotomy incision was closed using a combination of absorbable sutures (Ethicon) and wound clips (World Precision Instruments), which were removed 7 days post-surgery.

Bromodeoxyuridine (BrdU) (Sigma, UK; 10mM (10μl/g weight of 10 mg/ml BrdU) in PBS) was injected intraperitoneally at the time of surgery with additional application 24 hours post surgery.

Transplanted *R26R-YFP/YFP* mice were checked periodically following surgery and of those transplanted 4 (of 66) were in obvious discomfort in the 12 hours following surgery and were euthanised according to protocol. The cause was determined to be perforation of the bowel. The rest were typically maintained for around 4 weeks post-transplantation before sacrifice and removal of bowel for analysis, although some animals were maintained up to 24 months to obtain long-term safety data. Three of the 5 transplanted Ednrb^tm1Ywa^ mutants developed abdominal swelling and loss of condition without obvious intestinal perforation 2–4 days after transplant and were euthanized. The other 2 animals were euthanized at day 5 post-transplant.

### PCR

In order to ascertain whether *Wnt1-cre;R26R-YFP/YFP*-derived transplanted cells migrated to locations other than the gut following transplantation into *R26R-YFP/YFP* recipients, PCR was performed to identify the cre transgene. Brain, lungs, heart, liver, spleen, kidneys, adrenal glands and gut mesentery were collected on sacrifice and frozen. *Wnt1-cre;R26R-YFP/YFP* positive control tissue was obtained from *Wnt1-cre;R26R*^*YFP/YFP*^ gut tissue, transplanted gut, YFP+ neurospheres and a *Wnt1-cre;R26R*^*YFP/YFP*^ ear biopsy. DNA was extracted using DNAReleasy (Anachem) diluted 1μl in 4μl water and heated to 75°C for 5min followed by 96°C for 2 min. PCR was performed using cre primers (5’-ACCCTGATCCTGGCAATTTCGGC and 5’-GATGCAACGAGTGATGAGGTTCGC) and a cycle with a 60°C annealing temperature.

### Immunohistochemistry

Cells and transplanted bowel were fixed and analysed using immunohistochemistry as described previously[24, 40]. The primary and secondary antibodies used in the study are listed in Table 1 and Table 2.

**Table 1:** Primary antibodies for immunohistochemistry studies.

PRIMARY ANTIBODY	CONCENTRATION	COMPANY
Mouse anti-TuJ1	1:500	Covance
Mouse anti-GFAP	1:500	Dako
Rabbit anti-GFP	1:500	Invitrogen
Mouse anti-GFP	1:500	Invitrogen
Chicken anti-GFP	1:500	Abcam
Goat anti-Sox10	1:300	Santa Cruz
Rabbit anti-S100	1:400	Dako
Rabbit anti-nNOS	1:400	Invitrogen
Rabbit anti-VIP	1:400	AbD Serotec
Goat anti-ChAT	1:300	Millipore
Rat anti-BrdU	1:20	Oxford Biotech
Mouse anti-Synaptophysin	1:250	AbD Serotec

**Table 2:** Secondary antibodies for immunohistochemistry studies.

SECONDARY ANTIBODY	ALEXA FLUOR	CONCENTRATION	COMPANY
Goat anti-rabbit	488	1:500	Invitrogen
Goat anti-mouse	488	1:500	Invitrogen
Anti-chicken	488	1:500	Abcam
Anti-mouse	568	1:500	Invitrogen
Anti-rabbit	568	1:500	Invitrogen
Anti-mouse	647	1:500	Invitrogen
Anti-rabbit	647	1:500	Invitrogen

Briefly, for wholemount immunolabelling, gastrointestinal tracts were fixed in 4% PFA (1h), washed (2x10min, PBS) and then blocked (1h –overnight, wholemount blocking solution (WBS) (PBS, 2% Triton X-100, 10% sheep serum). Primary antibodies were applied for 48h at 4°C before washing (2x1h, PBS with 1% Triton X-100) and addition of secondary antibodies (24h, 4°C). Secondary antibodies were washed and samples mounted on glass coverslips for imaging.

For labelling with BrdU antibody, samples were first labelled with other primary antibodies as described above. Samples were post fixed (4% PFA,10 min, RT) and washed 3x in PBS. This was followed by treatment with 2M HCl for 15minutes at RT. After washing (3x15min), Rat anti-BrdU (Oxford Biotech, UK, 1:20) was applied overnight at 4°C, followed by goat anti-rat 568 (1:500) secondary antibody. Post antibody washings and mounting were carried out as described above.

Images were acquired on a Zeiss LSM 710 confocal microscope (Zeiss, Cambridge, UK) and were processed using ImageJ and Adobe Photoshop software[41, 42].

### Calcium imaging of transplanted mouse ENCCs

Colonic segments were removed from transplanted mice as described and immediately immersed in previously oxygenated (95% oxygen/5% carbon dioxide) Krebs solution (in mM: 120.9 NaCl, 5.9 KCl, 1.2 MgCl2, 2.5 CaCl2, 11.5 glucose, 14.4 NaHCO3 and 1.2 NaH2PO4). After removing the mucosa, gut preparations were pinned tightly, serosal side up, in a Sylgard-lined chamber. Transplanted YFP+ cells were identified and imaged at this stage.

Tissues were then loaded with the fluorescent Ca^2+^ indicator Fluo-4AM (Molecular Probes, Invitrogen; 5mM) and Cremophor EL (Fluka Chemika, Buchs, Switzerland; 0.01%) in Krebs solution at room temperature for 20 minutes with continuous oxygenation.

After loading, tissues were washed (2x10 min, Krebs) prior to imaging. Live fluorescence imaging was performed on a Zeiss Examiner microscope equipped with a 20x (NA 1) water dipping lens, Poly V monochromator (TILL Photonics, Gräfelfing, Germany) and cooled CCD camera (Imago QE; TILL Photonics, Gräfelfing, Germany). The experimental chamber volume was maintained at 3ml via a gravity-fed perfusion system ensuring continuous perfusion (1ml/min) with 95% oxygen/5% carbon dioxide-gassed Krebs solution (at RT), excess solution was removed via a peristaltic suction pump. Fluo-4 was excited at 475nm, and its fluorescence emission was collected at 525/50 nm. Images (640X512 pixels^2^) were acquired at 2 Hz.

Electrical train stimulation (2 s, 20 Hz of 300 μs electrical pulses; Grass Instruments, Quincy, Massachusetts, USA) was applied via a platinum electrode (diameter 25 μm), inserted at a distance of 200μm from the most peripheral YFP+ cell or fibre in the observed transplanted region of interest. Care was taken during this insertion to ensure contact was restricted to endogenous (non YFP+) fibres. To confirm the neuronal origin of the signal in YFP+ transplanted cells, experiments were conducted in the presence of tetrodotoxin (1 μM, Sigma, Bornem, Belgium). Local application of high K^+^ was also used as a means of determining basic functionality of transplanted cells within host tissues.

Images were collected using TILLVision software (TILL Photonics) and post acquisition analysis performed in IGOR PRO (Wavemetrics, Lake Oswego, Oregon, USA). Movement artifacts were removed by registering the image stack to the first image. Regions of interest (ROI) were drawn over each cell, fluorescence intensity was normalised to basal fluorescence for each ROI(F/F0), and peaks analysed [43–45].

## Results

### YFP+ ENCCs form neurospheres containing neurons, glia and presumptive enteric neural stem cells

**Fig 1:**
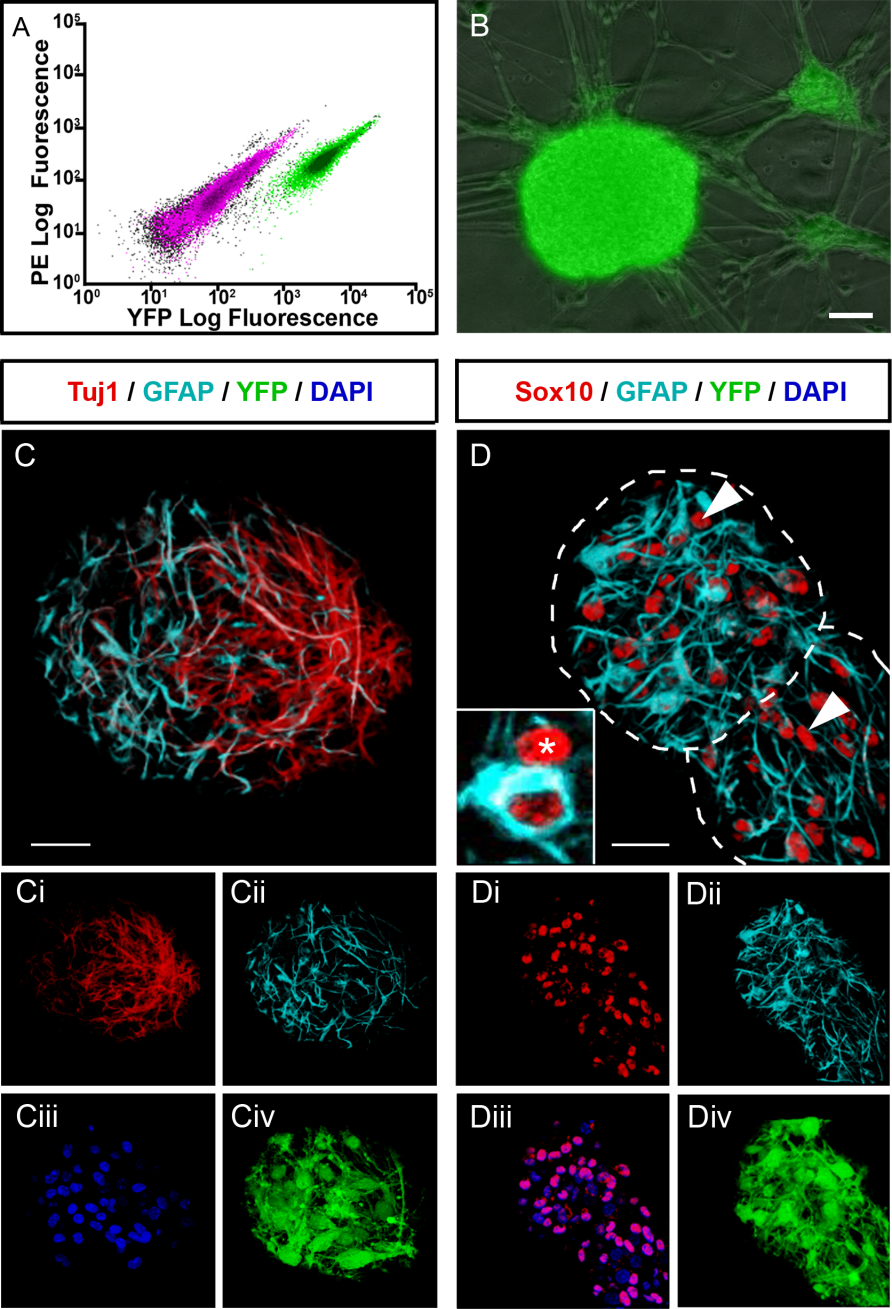
Isolation and culture of YFP+ enteric neural crest cells (ENCC) from postnatal Wnt1-cre;R26RYFP/YFP mouse gut. **A.** Flow cytometry profile showing the YFP+ cell population (green) separate from the enteric cell population (pink). **B.** Selected YFP+ cells form YFP+ neurospheres after 7 days in culture. **C.** Immunohistochemical analysis confirms that all cells within the neurospheres express YFP (green; **Civ, Div**). Neurospheres contain cells immunopositive for the neuronal marker TuJ1 (red; **C, Ci**) and the glial marker GFAP (cyan; **C, Cii**). DAPI labels nuclei in blue (Ciii)**. D.** Neurospheres (outlined by dotted lines) also contain cells (asterisk and arrowheads) that express Sox10 (red; **D, Di** and **Diii,** arrowheads) but are negative for GFAP (cyan; **D, Dii**) i.e. presumptive ENSSCs. Inset in **D** shows a cross section through a Sox10+/GFAP- cell (red only; asterisk; presumptive ENSSC) adjacent to a Sox10+/GFAP+ cell (red and cyan; presumptive glial cell). Scale bar in **B-D** = 20μm.

Intestinal muscle strips were taken from *Wnt1-cre;R26R-YFP/YFP* mice aged P2-4 in which NCC and their derivatives express YFP. Following gut dissociation, YFP+ ENCCs were selected by FACS where these cells accounted for 16.5±1.9% (n = 6) of the total gut cell population (Fig 1A). Selected YFP+ mouse ENCCs formed characteristic neurospheres of approximately 20μm in diameter within 2 weeks in culture, which increased in size to approximately 120μm within a month (Fig 1B). The neurospheres comprised cells expressing ENS markers such as the pan-neuronal marker TuJ1 (31.7%±3.2% of cells within the neurosphere (n = 3)) and the glial marker GFAP (27.7%±5.9% (n = 3)), which maintained their YFP expression throughout culture (Fig 1C). They also contained cells that were immunopositive for Sox10 but negative for GFAP (Fig 1D), the expression profile of presumptive enteric neural stem cells (Sox10+ cells accounted for 67.7%±8.7% of cells within the neurosphere (n = 3), thus Sox10+;GFAP- enteric neural stem cells accounting for around 40%).

### Transplanted ENCCs show appropriate colonisation, localisation and formation of ENS-like networks in ganglionic host gut

**Fig 2:**
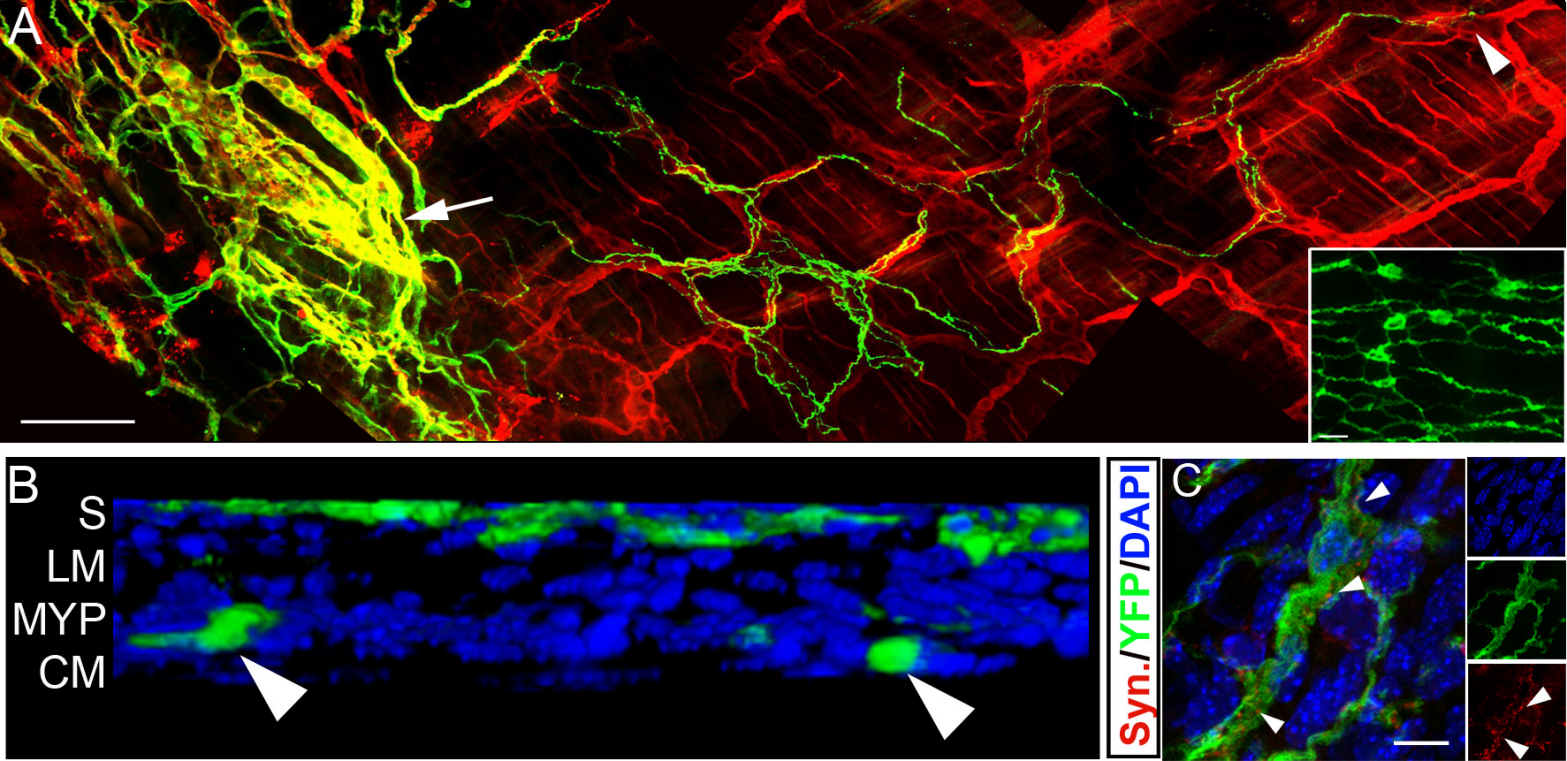
ENCCs from transplanted murine neurospheres show appropriate colonisation, localisation and formation of ENS-like networks in recipient wild-type gut. **A.** Wholemount gut preparation showing YFP+ transplanted cells (green) projecting along endogenous TuJ1+ (red) ENS nerve fibres. Arrow indicates cell bodies at the presumptive site of transplantation and arrowhead indicates distal extent of projections of transplanted cells. YFP+ transplanted cells also expressing the neuronal marker TuJ1 are seen as yellow. Inset shows a high power image taken from boxed region in S1 Fig panel A revealing interconnections and network formation between YFP+ cell bodies. **B.** Confocal 3D reconstruction showing YFP+ transplanted cells (green) located at the site of transplantation on the serosal surface (S) and within the myenteric plexus (MYP; arrowheads) between the inner circular (CM) and outer longitudinal (LM) muscle layers. **C.** YFP+ transplanted cells (green) co-locate with the synaptic marker synaptophysin (red; arrowheads). Scale bar in **A** = 100μm; **C** = 25μm. Inset in C shows individual channels.

YFP+ neurospheres were transplanted into postnatal wild-type mouse distal hindgut. YFP+ transplanted ENCCs were subsequently identified within the gut wall of 56/62 animals examined (90.3%), up to 24 months post-transplantation. Within the gut, YFP+ ENCCs spread orally and anally from the site of transplantation and formed extensive branching networks co-located with the endogenous ENS (Fig 2A including inset and S1 Fig panel A). By 15 weeks post-transplantation YFP+ networks, on average, covered an area of 4.3±3.1mm^2^ and cell bodies were observed at a distance of 1.4±0.4mm from the site of transplantation (n = 10) (S1 Fig panel B and C). These parameters, as well as the proximal-distal spread of transplanted cells, showed positive correlation to the number of days post-transplantation (S1 Fig panel B (r = 0.68, n = 32, p<0.01); 1C (r = 0.54, n = 28, p<0.01); and 1D (r = 0.51, n = 28 p<0.01) respectively). There was no significant difference between the mean oral (0.9±0.38mm) and aboral (0.9±0.4mm) spread (t = 0.45, n = 6, p<0.5).

Cell bodies (Fig 2B) of, and projections (arrowhead, Fig 2A) from, transplanted YFP+TuJ1+ neuronal cells were located within the endogenous myenteric plexus and projected within it for several millimetres. Punctate labelling around transplanted cells with the synaptic vesicle protein synaptophysin (arrowheads, Fig 2C) suggested synapse formation within the host gut.

### Transplanted YFP+ ENCCs show functional integration within host gut

**Fig 3:**
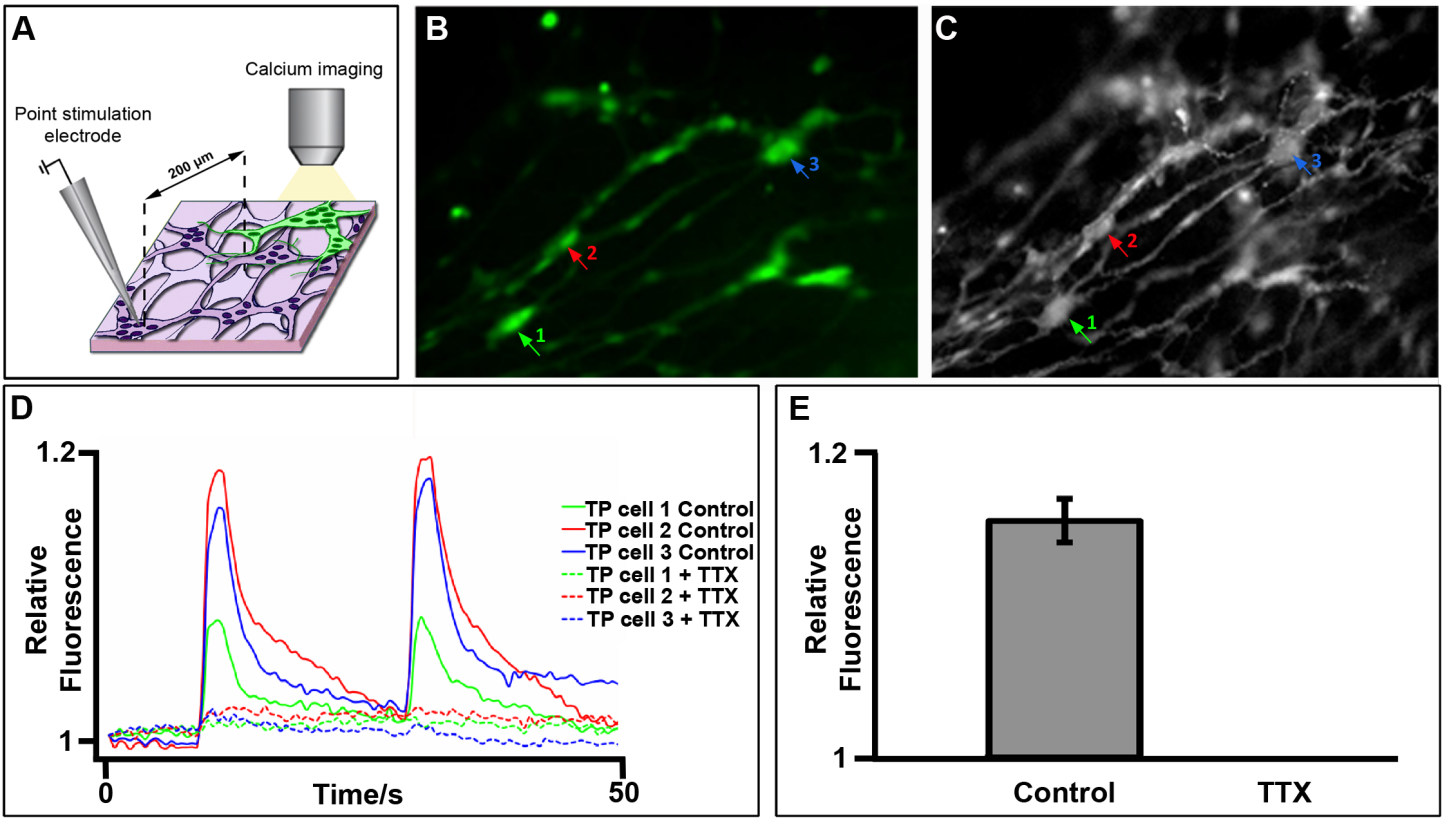
Transplanted YFP+ cells show functional integration within host gut. **A.** Schematic of experimental protocol demonstrating electrical point stimulation of host enteric nerve fiber at a site distant from YFP+ transplanted cells. **B,C.** Representative images of YFP+ cells before (**B**) and after (**C**) Fluo4-AM loading. Arrows indicate transplanted neurons (TP cell) from which Ca^2+^ responses are plotted in **(D). D.** Representative traces showing Ca^2+^ responses recorded as F/F0 from TP cell in control conditions (solid lines) and after addition of TTX (dotted lines). **E.** Summary data demonstrating abolition of Ca^2+^ responses in the presence of TTX (43 cells, n = 6). Also see S1 Movie.

In order to test the functional integration of transplanted YFP+ ENCCs within the host gut musculature, we examined [Ca^2+^]_i_ upon electrical stimulation of the endogenous enteric neural network (Fig 3A) or used application of high K^+^ as a means of neuronal activation. Electrical point stimulation of endogenous enteric nerve fibres resulted in calcium transients (F/F0 = 1.16±0.01; 43 cells, n = 6) in both the cell bodies and fibres of YFP+ transplanted ENCCs (Fig 3B–3D and S1 Movie). These calcium transients were abolished in the presence of 1μM TTX, confirming their neuronal identity (Fig 3D and 3E; 43 cells, n = 6) (S1 Movie). Local application of high K^+^ also resulted in similar widespread calcium transients throughout YFP+ transplanted cell networks and contributed to large contractions of the musculature (data not shown).

### Transplanted YFP+ ENCCs generate a range of ENS cell types in vivo

**Fig 4:**
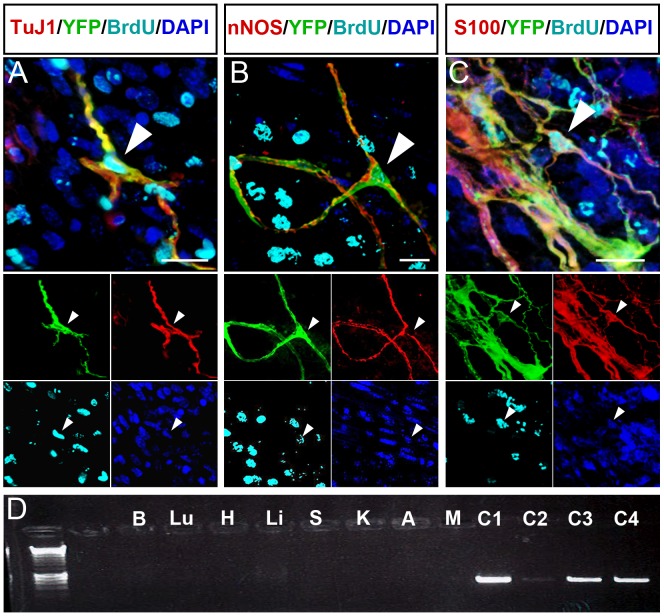
Transplanted YFP+ mouse ENCCs proliferate and generate enteric neurons and glia but do not spread beyond transplanted gut. **A**-**C.** Z-projections in which transplanted YFP+ cells co-express ENS markers (yellow; arrowheads) including the pan-neuronal marker TuJ1 (**A**), the inhibitory neuronal marker nNOS (**B**), and the glial marker S100 (**C**). Transplanted cells co-expressing ENS markers also demonstrate BrdU incorporation (cyan; arrowheads; **A-C** and insets). DAPI labels nuclei in blue (**A-C** and insets). Insets show individual channels. **D.** *Wnt1-cre;R26R*^*YFP/YFP*^ expressing transplanted cells (YFP+) were identified within *R26R*^*YFP/YFP*^ recipient mice by the presence of the cre transgene on PCR. Representative cre-PCR to identify cre expressing cells within the major organs from a transplanted mouse. Brain (B), lungs (Lu), heart (H), liver (Li), spleen (S), kidneys (K), adrenal glands (A) and gut mesentery (M) were all negative for cre. Control tissues: *Wnt1-cre;R26R*^*YFP/YFP*^ gut tissue (C1), transplanted gut (C2), YFP+ neurospheres (C3) and *Wnt1-cre;R26R*^*YFP/YFP*^ ear biopsy (C4) are cre+. Scale bar in **A-C** = 20μm.

The ENS cell-types of transplanted YFP+ ENCCs were analysed by immunofluorescence 4-weeks after transplantation. 53±14% of transplanted cells expressed the neuronal marker TuJ1 (n = 13) (Fig 4A and S2 Fig panel A and S3 Fig panel A). nNOS expressing cells accounted for the majority of neurons (50±5% of transplanted cells; Fig 4B and S2 Fig panel B and S3 Fig panel A). Transplanted cells expressed other neuronal markers, such as ChAT, Calbindin and VIP (S2 Fig panels C, D and E respectively) but these cell types were less numerous (data not shown). Transplanted cells also expressed the glial cell markers S100 and GFAP (Fig 4C and S2 Fig panel F and G). S100+ cells comprised 64±22% of transplanted cells (S3 Fig panel A; n = 3).

### YFP+ ENCCs proliferate post-transplant giving rise to enteric neurons and glia

The proliferative capability of transplanted YFP+ cells was assessed by BrdU incorporation. BrdU pulses were administered at transplantation and 24h later. BrdU incorporation was identified within YFP+ cells and quantified in different cell types (S3 Fig panel B). 26±19% of TuJ1+ cells (Fig 4A and S3 Fig panels B and C; n = 13), 36±19% of nNOS+ cells (Fig 4B and S3 Fig panel B, n = 3) and 32±17% of S100+ cells (Fig 4C and S3 Fig panel B; n = 3) showed BrdU incorporation. This suggests that varying proportions of transplanted cells expressing neuronal and glia markers are derived from cells that proliferate during the 48hrs following transplantation.

### Transplanted YFP+ ENCCs do not show uncontrolled proliferation, form tumours or spread to other organs

Long-term studies of mice that were the recipients of YFP+ ENCC transplants were conducted to assess safety of these transplants. Although initial BrdU exposure (48 hours after transplantation) resulted in BrdU incorporation in transplanted cells, exposure 48hrs prior to culling at 4 weeks post-transplantation did not show BrdU incorporation (data not shown) suggesting there was no uncontrolled proliferation of transplanted cells. Macroscopic and PCR examination of transplanted animals and tissues aged 19–25 months (including brain, lungs, heart, liver, spleen, kidneys, adrenal glands and gut mesentery) failed to identify any YFP fluorescence or *cre* transgene within organs other than the gut and positive controls (Fig 4D).

### YFP+ ENCCs transplanted into aganglionic Ednrb^tm1Ywa^ gut form branching neuronal networks

**Fig 5:**
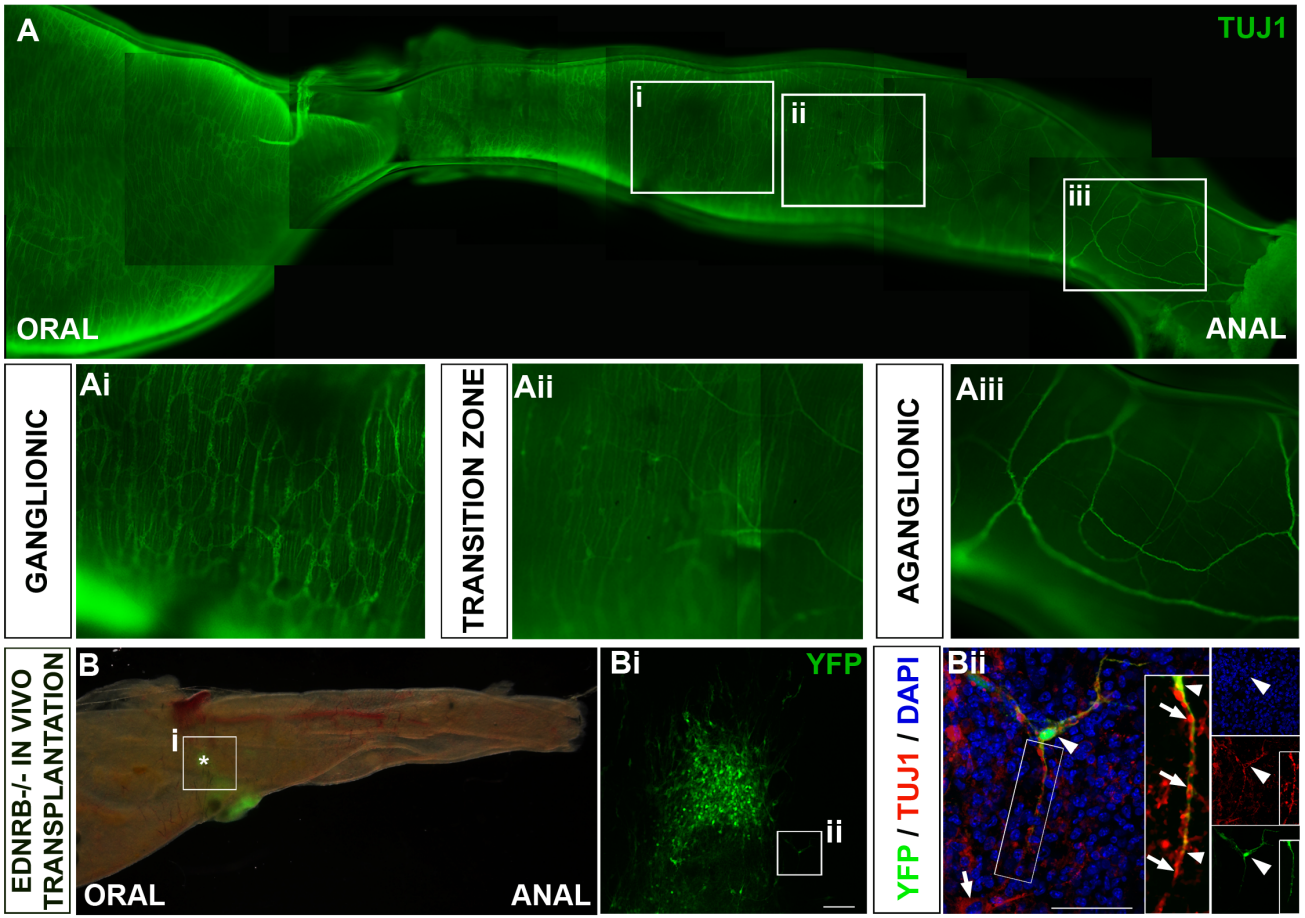
YFP+ ENCC colonise aganglionic Ednrb^tm1Ywa^ gut in vivo. **A.** Neurons in an Ednrb^tm1Ywa^ colon immunolabelled with TuJ1 progressing from proximal ganglionated gut (**Ai**), through the partially ganglionated transition zone (**Aii**) to the distal aganglionic gut (**Aiii**). **B.** Ednrb^tm1Ywa^ gut 3 days after transplantation with YFP+ ENCCs (green) which could be identified in distal colon. Boxed area is area enlarged in Bi and asterisk denoted presumptive site of transplantation in this region. **Bi**. Interconnections are visible between transplanted cells that have spread from the site of transplantation. **Bii.** Z-projections in which endogenous neurons express the neuronal marker Tuj1 (red; arrow) and YFP+ transplanted cells (green) also coexpress Tuj1 (yellow; arrowhead). Projections from YFP+;Tuj1+ transplanted cells closely associate with projections from endogenous Tuj1+ neurons present in the transition zone (insets in **Bii;** arrowheads and arrows respectively). Scale bar in **Bi** = 100μm; **Bii** = 50μm. DAPi is shown in blue.

Ednrb^tm1Ywa^ mice are characterised by a variable length of aganglionosis in the distal colon and mutants within our colony survive for around two weeks. Tuj1 immunohistochemistry showed the proximal colon appeared normally ganglionated (Fig 5A and 5Ai) progressing distally to the hypoganglionated region of transition zone (Fig 5A and 5Aii) and to the distal-most bowel, which, although showing innervation by extrinsic fibres, lacks an intrinsic ENS (Fig 5A and 5Aiii).

YFP+ neurospheres were transplanted into the most distal portion of hindgut accessible from the peritoneal cavity of Ednrb^tm1Ywa^ mice (n = 5). Transplanted cells survived and could be identified by their YFP fluorescence in 4 out of 5 guts after 3–5 days (Fig 5B). Transplanted cells included both Tuj1+ neurons (Fig 5Bii) and S100+ glia (data not shown) and had spread out to form interconnected neural networks (Fig 5Bi). Furthermore projections from transplanted neuronal cells associated closely with the endogenous neurons of the transition zone (Fig 5Bii and inset in 5Bii).

## Discussion

Our studies confirm the feasibility of the *in vivo* transplantation of post-natally sourced ENCCs into both ganglionic and aganglionic post-natal intestine. Not only are transplanted cells able to engraft successfully into significant segments of intestine they are able to show critical functional integration with the endogenous neural networks. Importantly, our work suggests that ENCC transplantation is safe in the long-term.

Only one other study to date has robustly tested engraftment and functional integrity of ENCCs following *in vivo* transplantation [32]. Our study, however, extended this work significantly across a number of domains from assessment of engraftment, post-transplant cell behaviour and functional integration with endogenous neurons, through to long-term studies of viability and safety. We were also able to assess *in vivo* transplantation into aganglionic intestine.

Our exclusive use of postnatal intestine in the *in vivo* setting as both donor and recipient of ENCCs served to best recapitulate the clinical setting of autologous transplantation. We have previously shown that ENCCs can be harvested from human post-natal gut, even utilising minimally invasive techniques such as endoscopy. Such cells successfully colonised recipient aganglionic gut either embryonic gut maintained on chorioallantoic membrane or human gut maintained *in vitro* [24]. Although human ENCCs remain to be tested in the *in vivo* setting our murine studies confirm that the therapeutic strategy of autologous transplantation of ENCCs into aganglionic and euganglionic intestine is a viable option.

Previous studies have suggested that recipient intestine with established endogenous enteric neurons is poorly receptive to transplanted cells [10]. Our studies and those of others [16, 33, 35, 46] confirm this not to be the case opening up the considerable potential for ENCC therapy for a range of enteric neuropathies not characterised by aganglionosis such as slow transit constipation, intestinal pseudo-obstruction or those occurring following injury.

In our studies transplanted ENCCs showed significant proliferation in recipient intestine following transplantation. BrdU assays confirmed that although highly proliferative following delivery (giving rise to both neurons and glia in approximately equal proportions), transplanted cells did not continue to proliferate and seem to achieve a steady state within a few weeks. This mimics the behaviour of the endogenous ENS after insult or injury [47] implying that transplanted cells may be responding to endogenous signalling mechanisms tasked with maintaining the structural and functional integrity of the ENS, perhaps at the site of injury secondary to implantation of the neurospheres. Nonetheless, this capacity to expand until presumably a *status quo* is achieved holds huge promise given it suggests that sufficient ‘therapeutic’ cells may be generated following transplant to colonise and integrate within the recipient ENS.

Of course therapeutic success is reliant on appropriate differentiation and functional integration. In our studies, and as recently reported [32], we utilised YFP+ ENCCs derived from postnatal *Wnt1-cre;R26R-YFP/*YFP mice to generate YFP+ neurospheres, comprised of mature neurons and glia as well as enteric neural stem cells but devoid of non-ENS cells (e.g. smooth muscle and fibroblast-like cells). Upon transplantation, the YFP+ ENCCs were capable of generating both neurons and glia including a range of neuronal subtypes. Importantly nNOS+ cells were the predominant neuronal subtype evident in transplanted ENCCs. This may be critical for therapy of enteric neuropathies, many of which are characterised by loss of ENS or more specifically of inhibitory nNOS neurons with tonic contraction and a failure of adequate relaxation of the affected intestine[48, 49]. Our further studies will aim to determine whether the nNOS component of transplanted cells is capable of restoring the inhibitory response in models of nNOS deficiency

Transplanted ENCCs localised to the myenteric plexus and with their projections followed established networks for considerable distances, which, accompanied by expression of the synaptic marker synaptophysin, suggested integration with the host neuromusculature. In work by Hotta et al the authors made intracellular recordings of individual transplanted ENCCs confirming them to be functionally active neurons and suggesting their functional integration within recipient gut [35]. Using the different approach of intracellular calcium imaging we were able to further show that stimulation of endogenous enteric nerve fibres resulted in widespread calcium transients throughout multiple cells within YFP+ transplanted neural networks. Such firing of multiple transplanted cells suggests integration of circuitry, which would be required to impart functional improvements in models of neuropathy. This technique has been used widely to demonstrate ENS functionality [44, 45]. These studies do suggest functional integration of the transplanted networks with the endogenous neuromusculature but do not confirm that this translates to changes in colonic motility as measured by contractile function or indeed transit of luminal contents. The use of wild-type ganglionic intestine precludes this assessment given it is unlikely that one is able to see a supra-physiological change in motility. This will need to be done in the context of models of enteric neuropathy or aganglionosis.

To date studies demonstrating the colonisation of aganglionic gut by transplanted ENCCs have been almost completely limited to *in vitro* experiments [24, 28] given the very poor survival of the mouse models of Hirschsprung disease. We have been able to progress these studies into *in vivo* transplantation with data that supports the ability of ENCCs to rebuild neural networks. Although, the survival of recipient EDNRB null animals was limited we were able to deliver the cells *in vivo* and show that they were able to engraft successfully. Accepting a short duration for assessment transplanted ENCCs survived, spread out and formed close associations with endogenous neurons, providing promise that in a therapeutic setting transplanted ENCCs may be able to make the connections with the endogenous ENS required to make functional circuitry. Although, in keeping with the experience of others, our experiments continue to be hampered by the poor survival of mouse models of Hirschsprung Disease (including Ednrb and monoisoformic Ret51), novel strategies may facilitate future studies in such animals. Stamp et al recently report a surgical model in a rat model of Hirschsprung disease whereby formation of an intestinal stoma enabled good post-natal survival and well-being theoretically facilitating assessment of transplants[50]. Alternatives include the use of less affected models of enteric neuropathy, such as the nNOS null mutant, which is compatible with survival, but has detectable neurological deficits [51], or other models of aganglionosis such as chemical ablation of the ENS using treatments such as benzalkonium chloride (BAC) [47, 52].

A strength of our studies is the robust assessment of long-term safety up to 24 months post transplantation. We could consistently visualise transplanted YFP+ cells within hindguts of recipient mice in these end stage experiments, however we never observed YFP-derived tumours in any organ. Additionally PCR analysis demonstrated that transplanted cells were restricted to the distal colon with no evidence of spread or seeding to sites away from the target organ. This containment of transplanted cells taken together with their restricted proliferative capacity to the period immediately following transplantation provides critical safety data for the application of any future cellular therapy. Although we did not assess transplanted cells for genetic alterations our studies strongly support the long-term safety and suggest a minimal risk of malignant transformation of ENCC transplants or metastatic spread.

In conclusion, our findings demonstrate that within the context of *in vivo* transplantation postnatal ENCCs are able to engraft successfully and safely within recipient mouse bowel. These observations significantly support and advance the development of cell replacement strategies for a range of enteric neuropathies but this needs to be verified by further studies detailing *in vivo* transplantations into more robust models of these devastating disorders.

## Supporting Information

S1 Fig Spread of YFP+ mouse ENCC following transplantation into *in vivo* gut. **A.** YFP+ transplanted cells migrate from the presumptive site of transplantation (asterisk) to form branching networks. Arrowheads indicate oral- and anal-most cells. **B.** Quantification of the area covered by networks of transplanted cells originating from an individual neurosphere plotted over time (days post-transplant), (R = 0.54; n = 28; p<0.01). **C.** Quantification of the maximal migration of transplanted YFP+ cells from the presumptive site of transplantation plotted as a function of time (days post-transplant) (R = 0.68; n = 32; p<0.01). **D.** Quantification of the maximal proximal-distal spread of transplanted YFP+ cells plotted as a function of time (days post-transplant) (R = 0.51; n = 28; p<0.01). Scale bar in **A** = 250μm.
(TIF)

S2 Fig Transplanted YFP+ ENCC generate neurons and glia, including different neuronal subtypes. **A-G.** 3D reconstructions (low and high magnification) of z-stacks taken from wholemount gut preparations in which YFP+ transplanted cells (green) are immunohistochemically labelled with a range of ENS markers (red; co-expression yellow and arrowheads). Transplanted cells express the pan neuronal marker TuJ1 (**A**), inhibitory neuronal markers nNOS and VIP (**B, E**), excitatory neuronal markers ChAT and Calbindin (**C, D**), and the glial markers S100 and GFAP (**F, G**). DAPI labels nuclei in blue. Scale bar in **A-G** low magnification = 50μm; high magnification = 10μm. Insets show individual channels.
(TIF)

S3 Fig Proliferation and safety data for transplanted YFP+ mouse ENCC. **A.** Percentage of TuJ1+, nNOS+ and S100+ cells in the total population of transplanted cells (n = 13, 3, 3 respectively). **B.** Percentage of TuJ1+, nNOS+ and S100+ transplanted cells showing BrdU incorporation (n = 13, 3, 3 respectively). **C.** Percentage of TuJ1+ transplanted cells showing BrdU incorporation with high inter-sample variability, but low within-sample variability.
(TIF)

S1 Movie Ca^2+^ responses of transplanted YFP+ mouse neurons following electrical stimulation of endogenous ENS. Representative videos of Ca^2+^ responses following point stimulation of the endogenous ENS. Left panel shows activation of transplanted YFP+ cells in control conditions. In the presence of 1μM TTX, evoked Ca^2+^ responses are abolished (middle panel) and are restored after washout (right panel). Equivalent transplanted cells (Fig 2B), Ca^2+^ response traces (Fig 2D) and cumulative data plots (Fig 2E) are presented in the main manuscript.
(AVI)
