# Supplementary figures and images for: In Vivo Transplantation of Enteric Neural Crest Cells into Mouse Gut; Engraftment, Functional Integration and Long-Term Safety
Source: PLoS One. 2016 Jan 29;11(1):e0147989. doi: 10.1371/journal.pone.0147989 (PMC4732685; doi:10.1371/journal.pone.0147989)

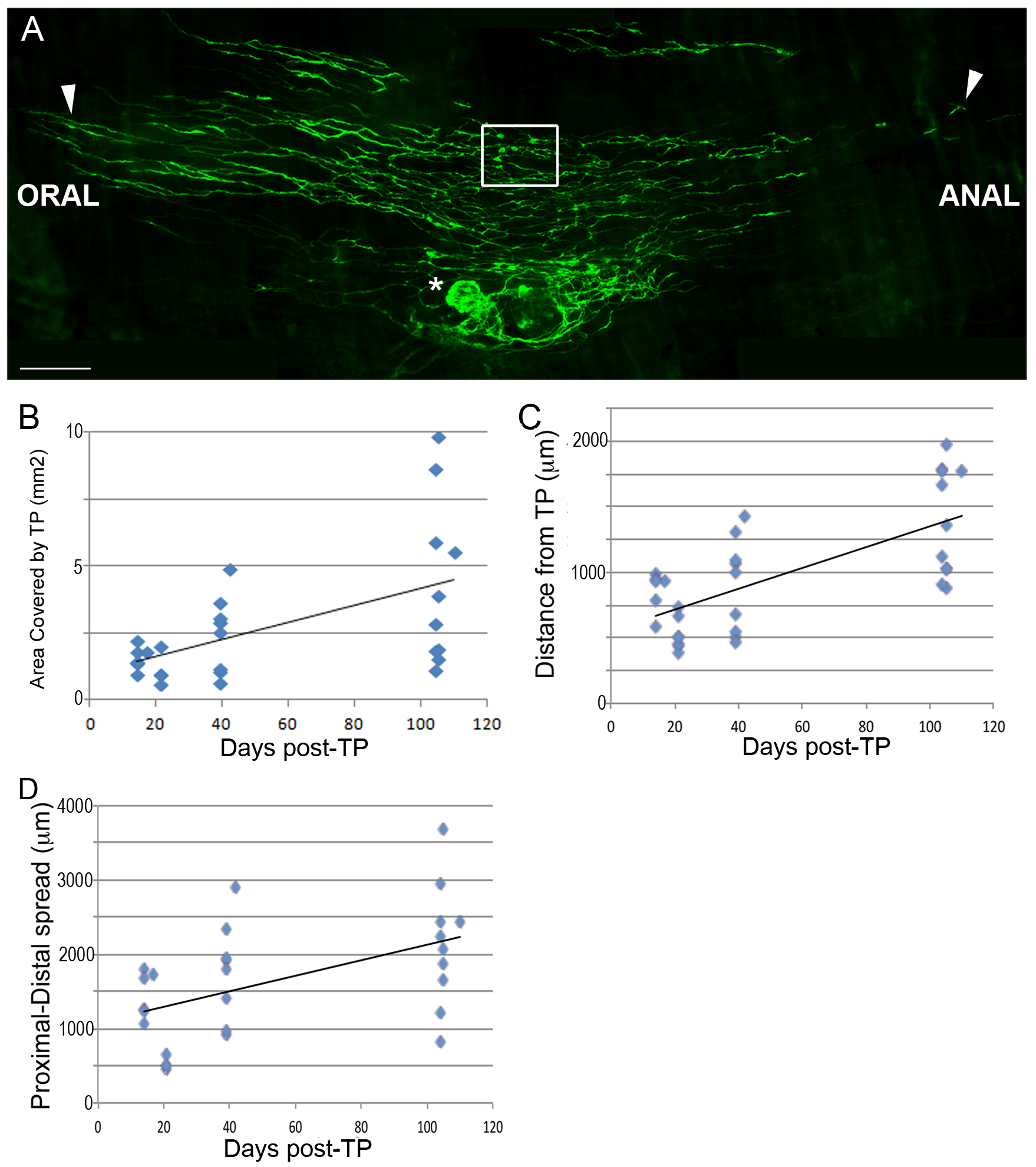

Supplement: S1 Fig — A. YFP+ transplanted cells migrate from the presumptive site of transplantation (asterisk) to form branching networks. Arrowheads indicate oral- and anal-most cells. B. Quantification of the area covered by networks of transplanted cells originating from an individual neurosphere plotted over time (days post-transplant), (R = 0.54; n = 28; p<0.01). C. Quantification of the maximal migration of transplanted YFP+ cells from the presumptive site of transplantation plotted as a function of time (days post-transplant) (R = 0.68; n = 32; p<0.01). D. Quantification of the maximal proximal-distal spread of transplanted YFP+ cells plotted as a function of time (days post-transplant) (R = 0.51; n = 28; p<0.01). Scale bar in A = 250μm. (TIF) [file pone.0147989.s001.tif]

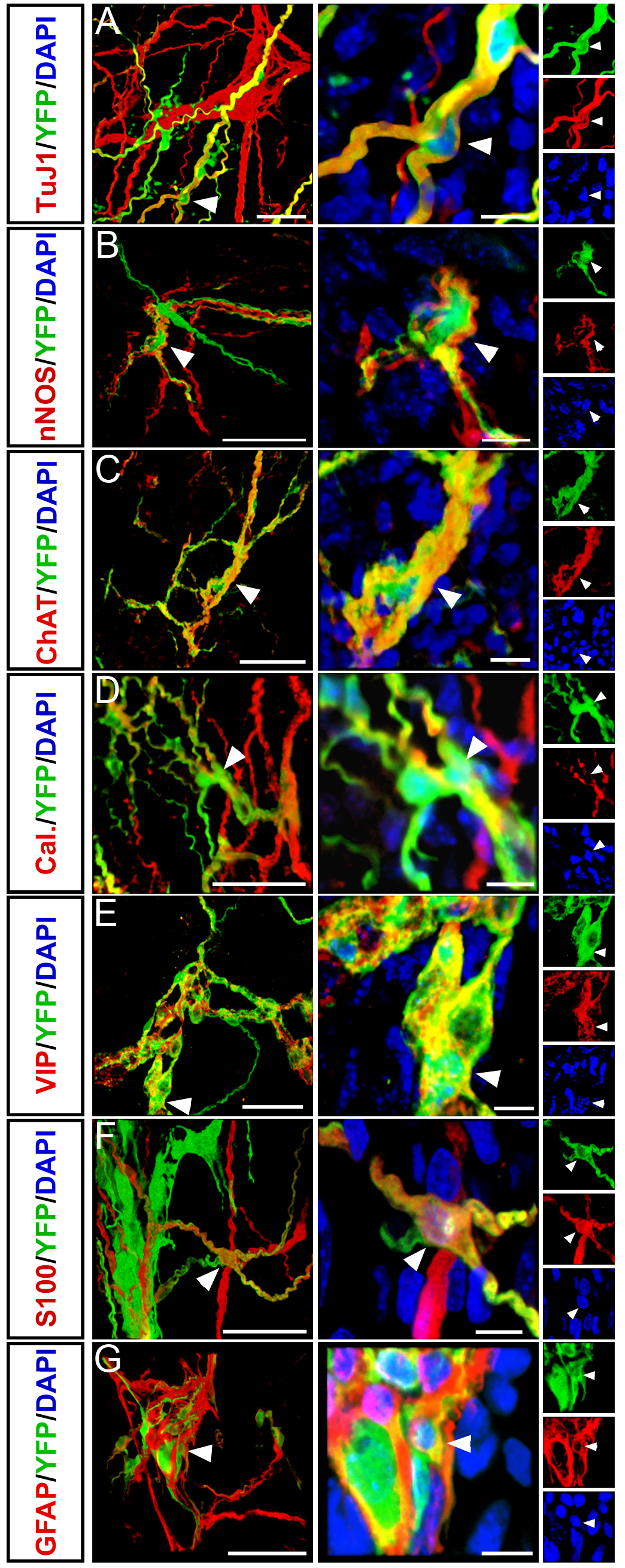

Supplement: S2 Fig — A-G. 3D reconstructions (low and high magnification) of z-stacks taken from wholemount gut preparations in which YFP+ transplanted cells (green) are immunohistochemically labelled with a range of ENS markers (red; co-expression yellow and arrowheads). Transplanted cells express the pan neuronal marker TuJ1 (A), inhibitory neuronal markers nNOS and VIP (B, E), excitatory neuronal markers ChAT and Calbindin (C, D), and the glial markers S100 and GFAP (F, G). DAPI labels nuclei in blue. Scale bar in A-G low magnification = 50μm; high magnification = 10μm. Insets show individual channels. (TIF) [file pone.0147989.s002.tif]

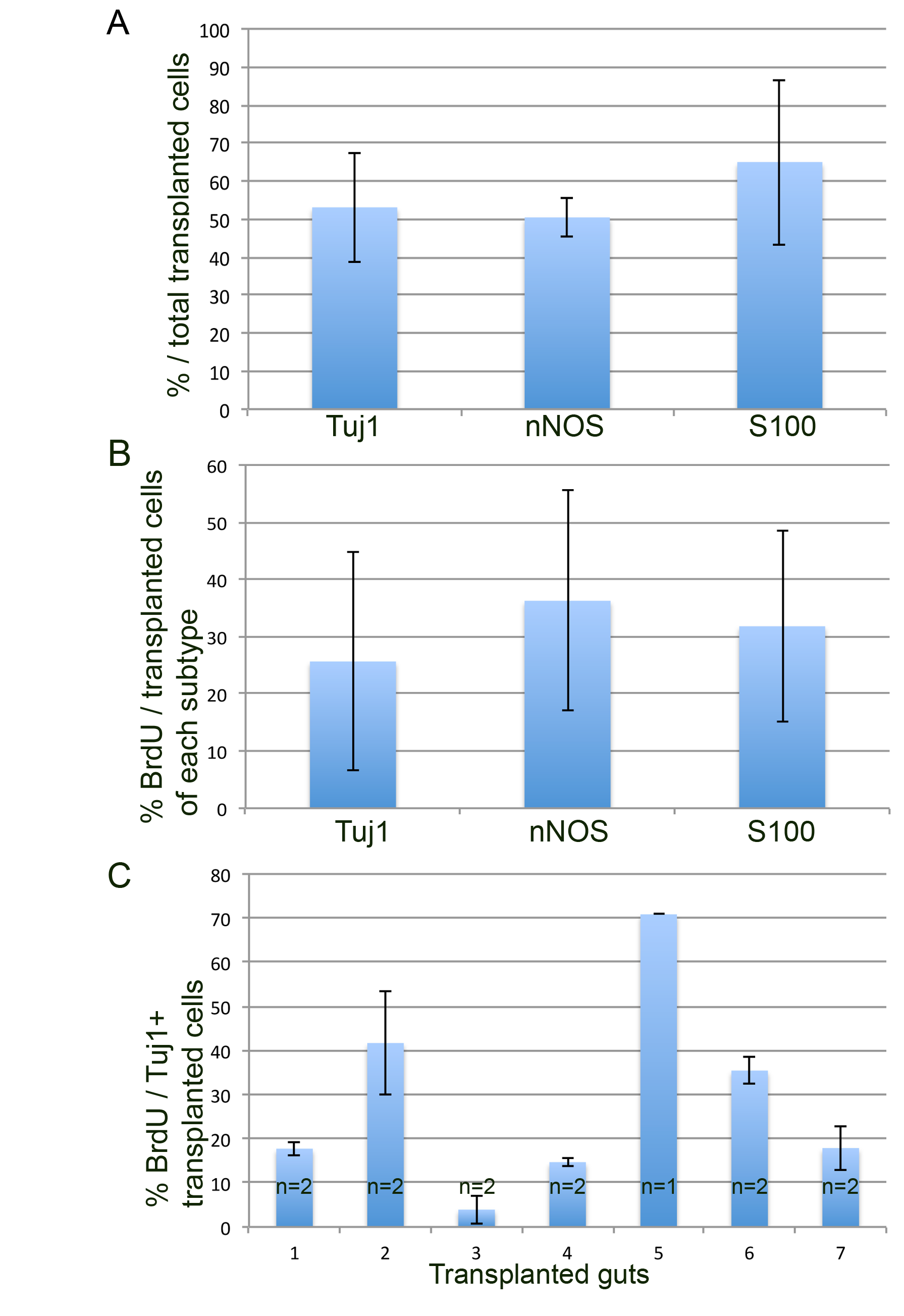

Supplement: S3 Fig — A. Percentage of TuJ1+, nNOS+ and S100+ cells in the total population of transplanted cells (n = 13, 3, 3 respectively). B. Percentage of TuJ1+, nNOS+ and S100+ transplanted cells showing BrdU incorporation (n = 13, 3, 3 respectively). C. Percentage of TuJ1+ transplanted cells showing BrdU incorporation with high inter-sample variability, but low within-sample variability. (TIF) [file pone.0147989.s003.tif]
